# Effectiveness of tobacco control television advertising in changing tobacco use in England: a population‐based cross‐sectional study

**DOI:** 10.1111/add.12501

**Published:** 2014-03-10

**Authors:** Michelle Sims, Ruth Salway, Tessa Langley, Sarah Lewis, Ann McNeill, Lisa Szatkowski, Anna B. Gilmore

**Affiliations:** ^1^ UK Centre for Tobacco and Alcohol StudiesDepartment for HealthUniversity of BathBathUK; ^2^ Department for HealthUniversity of BathBathUK; ^3^ UK Centre for Tobacco and Alcohol StudiesDivision of Epidemiology and Public HealthUniversity of NottinghamNottinghamUK; ^4^ UK Centre for Tobacco and Alcohol StudiesNational Addiction CentreInstitute of PsychiatryKing's College LondonLondonUK

**Keywords:** Cigarettes, consumption, gross rating points (GRPs), mass media campaign, smoking prevalence, smoking rates, television advertisement, tobacco control

## Abstract

**Aim:**

To examine whether government‐funded tobacco control television advertising shown in England between 2002 and 2010 reduced adult smoking prevalence and cigarette consumption.

**Design:**

Analysis of monthly cross‐sectional surveys using generalised additive models.

**Setting:**

England.

**Participants:**

More than 80 000 adults aged 18 years or over living in England and interviewed in the Opinions and Lifestyle Survey.

**Measurements:**

Current smoking status, smokers' daily cigarette consumption, tobacco control gross rating points (GRPs—a measure of per capita advertising exposure combining reach and frequency), cigarette costliness, tobacco control activity, socio‐demographic variables.

**Findings:**

After adjusting for other tobacco control policies, cigarette costliness and individual characteristics, we found that a 400‐point increase in tobacco control GRPs per month, equivalent to all adults in the population seeing four advertisements per month (although actual individual‐level exposure varies according to TV exposure), was associated with 3% lower odds of smoking 2 months later [odds ratio (OR) = 0.97, 95% confidence interval (CI) = 0.95, 0.999] and accounted for 13.5% of the decline in smoking prevalence seen over this period. In smokers, a 400‐point increase in GRPs was associated with a 1.80% (95%CI = 0.47, 3.11) reduction in average cigarette consumption in the following month and accounted for 11.2% of the total decline in consumption over the period 2002–09.

**Conclusion:**

Government‐funded tobacco control television advertising shown in England between 2002 and 2010 was associated with reductions in smoking prevalence and smokers' cigarette consumption.

## Introduction

Most of the evidence in favour of the effectiveness of mass media campaigns (MMCs) in reducing adult smoking prevalence and cigarette consumption comes from studies evaluating campaigns run for short time‐periods in countries with little other tobacco control (TC) activity. These studies, conducted in the mid‐1970s to mid‐1990s 1, were either community‐level studies with intervention and control communities 2 or population‐level studies 7, with most focusing on television campaigns 2, generally considered the most powerful medium to appeal to mass audiences. Since the 2000s, many countries began to run large‐scale MMCs for extended periods of time as part of multi‐component national TC programmes, rendering the findings from these earlier studies of limited relevance. The methodological challenges inherent in studying the effectiveness of current MMCs 1 are complicated by the absence of a control group and the difficulty of disentangling the contribution of MMCs from other TC policies. World‐wide, no study has isolated the effectiveness of MMCs on smokers' cigarette consumption from other components of a comprehensive TC programme, and only two studies (in Australia and the United States) have achieved these using national smoking rates as the outcome variable 13. Advertising content differs between these countries, with Australia showing mainly the negative health impacts of smoking and the United States including anti‐industry messages. In contrast, the United Kingdom has not shown anti‐industry messages, focusing instead on both the negative health impacts of smoking and campaigns with positive messages about how to quit 15. The effectiveness of televised campaigns in the United Kingdom may therefore be different to findings elsewhere. Given the dearth of evidence in this area, it is vital that public health interventions such as this are evaluated in other countries to ensure that there will be evidence‐based decisions on whether or not these interventions are maintained.

In the United Kingdom, the effectiveness of TC MMCs on smoking prevalence and consumption has been evaluated only for short‐term campaigns targeting either a specific population subgroup 16 or region 3 in the 1990s, when there was little other TC activity. Since the publication of ‘Smoking Kills’ by the government in 1998 18, there has been a substantial increase in TC activity (Fig. S1 in Supporting information), including large‐scale TC MMCs, and since 2007 the United Kingdom has been identified as having the most comprehensive TC policies in Europe 19. However, there has been no evaluation of the effectiveness of these MMCs on smoking prevalence or cigarette consumption. In April 2010, the government froze spending on national public health campaigns in England. Campaigns were re‐introduced in September 2011, albeit at a lower level of funding 21, after a Department of Health report found that, following the funding cuts, quit attempts fell 22. The need for a more informed evidence base on MMCs has been highlighted in the government's 2011 TC marketing strategy 21 and a recent study showing the positive impact of such campaigns on calls to the National Health Service stop smoking helpline in England 23.

In this paper we evaluate whether government‐funded TC television advertising shown in England between 2002 and 2010 was associated with changes in smoking prevalence and cigarette consumption. Our focus is on television advertising, as this media channel accounts for both the major expenditure and exposure by far. This study will provide essential evidence for determining whether the cutting of government spending on MMCs is justifiable.

## Methods

### Population survey data

The Opinions and Lifestyle Survey (OS), a monthly cross‐sectional survey run by the Office for National Statistics 24, is designed to be representative of adults living in private households throughout Great Britain. Households are selected each month using a clustered, stratified, multi‐stage design, resulting in each address having an equal probability of selection. One adult aged 16 years and over is selected randomly from among all the over 16‐year‐olds in each household to be interviewed. We extracted data from monthly surveys conducted between 2002 and 2010. The number of months surveyed per year has changed over time, from a minimum of 6 months in 2003 to a maximum of 12 months in 2006 and 2008–2010.

Respondents were asked about their tobacco use. Smokers were defined as those answering ‘yes’ to the question: ‘Do you smoke cigarettes nowadays?’. Measures of cigarette consumption were based on responses to two questions: ‘How many cigarettes a day do you usually smoke at weekends?’ and ‘How many cigarettes a day do you usually smoke on weekdays?’. We derived an average number of cigarettes smoked per day by taking a weighted average of weekend and weekday consumption using weights of two‐sevenths and five‐sevenths, respectively (hereafter defined as average consumption). Respondents are also asked questions about their age, gender, government office region of residence (GOR), employment, education and gross income.

### Tobacco control score

A coding scheme was used to quantify TC activity in England each month. The scheme was based on four policies: (i) smoke‐free work and public places, (ii) bans on advertising and promotion, (iii) health warning labels on cigarette packets and (iv) treatment to help smokers stop. Scoring for each policy was identical to that assigned by the Tobacco Control Scale 26 to compare TC efforts across Europe (see Supporting information for further details). For each month, scores for each policy were summed to derive a total tobacco control score.

### Cigarette costliness

Cigarette costliness is defined as the ratio of the cigarette price to income in the same month. We measured price using the weighted average retail selling price of cigarettes (Box S1 in Supporting information) and income using monthly income reported by the OS respondent. Monthly income reported by respondents in the OS surveys represents their total gross income from all sources (earnings from employment and self‐employment, pension, state benefits, interest from savings and other sources such as rent) before deductions for income tax, National Insurance, etc.

### Advertising data

Gross rating points (GRPs) are a standard measure of average per‐capita advertising exposure and are commonly used in evaluations of televised MMCs 27. They combine reach and frequency and are equivalent to the summed ratings of individual advertisements [television ratings (TVRs)]. Television viewer figures at the time when the advertisements are shown are collected by the Broadcasters' Audience Research Board via a metered panel. We use total adult GRPs for all TC advertisements shown on television per month as an indicator of exposure to TC television advertising. For example, 400 GRPs per month indicates that, on average, 100% of the adult population were exposed to four advertisements per month, or 50% were exposed to eight advertisements, and so on. The GRP data used in this study relate to Department of Health‐funded campaigns shown in England from January 2002 until April 2010. During this period the Department of Health also funded Cancer Research UK and the British Heart Foundation to undertake media campaigns, and we also include GRP data from these campaigns. Together, these were the main purchasers of public sector TC advertising during this period.

### Design

Information on month and year of interview of the OS respondents aged 18 and over, living in England and interviewed in the OS between February 2002 and April 2010 inclusive (no OS survey conducted in January 2002) was used to match the survey data to information on GRPs, cigarette prices and the tobacco control score. Records with missing data on smoking status were excluded from both the smoking prevalence and cigarette consumption analyses (<0.05%). The analysis of cigarette consumption was restricted further to respondents who self‐reported as being a smoker. Both daily and non‐daily smokers were included. Records with missing consumption data were also excluded from this analysis (<0.4%).

### Statistical analysis—smoking prevalence

We modelled the relationship between GRPs and smoking prevalence using a binomial logistic generalized additive model. Given that we expected non‐linear relationships between some of the explanatory variables in the model and the outcome variable, this model allows a flexible specification of the dependence of the response on the covariates rather than restriction to parametric relationships.

As evidence suggests that TC media campaigns have their effect on smoking behaviour while campaigns are being broadcast and for a short time afterwards 9, we investigated the association between GRPs and smoking prevalence by including GRPs during the month of interview (an immediate effect), 1 month and 2 months earlier (i.e. lagged effects of 1 and 2 months) as three separate smooth terms using cubic regression splines. There was little correlation between these variables (Pearson's correlation coefficient in all two‐way comparisons was less than 0.22). The effective degrees of freedom (EDF) associated with a smooth term measures the degree of non‐linearity, with an EDF of 1 indicating that the shape of the relationship between the GRP term and the link function (log of the odds ratio of smoking) is linear.

To model the effect of other TC strategies, we included the tobacco control score as a categorical term and individual cigarette costliness as a cubic regression spline to allow for a non‐linear relationship. As monthly income reported by OS respondents was skewed strongly to the right, we log‐transformed cigarette costliness before analysis to make the distribution more symmetrical. All models also included the following individual‐level variables associated with smoking rates: cubic regression splines for age and income, and categorical variables for gender, GOR, education, employment status and social class (Table S2 in Supporting information). The number of individuals who declined to take part or could not be contacted in the OS survey rose slightly during the study period from approximately 30% to 40%. In order to compensate for non‐response, OS introduced non‐response weights based on age, sex and GOR of residence in April 2005. Rather than use these weights, which are only available from mid‐way through our study period, our inclusion of individual‐level variables for age, gender and GOR adjusted for non‐response bias over the entire study period.

As only one adult is interviewed per household in the OS survey, adults in households containing many adults have a lower chance of selection than those in households with few adults, and will therefore be under‐represented in the sample. Household size may have changed over the study period and be correlated with known determinants of tobacco use such as ethnicity or socio‐economic status (SES). We therefore included number of adults per household as a linear term to adjust for this unequal probability of selection.

### Statistical analysis—consumption

We modelled the relationship between GRPs and average cigarette consumption using a Poisson generalized additive model, and included the same covariates as in the prevalence model (Table S2 in Supporting information).

Overdispersion, indicating that there is more variability around the fitted values than is expected under a Poisson regression, was detected in the model. This could be an indicator of model misspecification (for example, missing covariates or interactions, outliers or non‐linear effects of covariates put in the model as linear terms) or evidence that real overdispersion exists (for example, the variability around the fitted values really is larger than expected under a Poisson regression and caused by clustering of observations). We included all major factors that determine tobacco use, considered non‐linear effects of covariates in our models and checked for outliers to minimize model misspecification, yet there was still a small amount of overdispersion detected. We therefore corrected the standard errors using a quasi‐Poisson model.

All models were fitted in R using the gam function from the library mgcv 30. All tests were two‐sided and performed at the 5% level of statistical significance. Although recent findings from Australia and the United States on the effectiveness of pharmaceutical advertisements for nicotine replacement therapy (NRT) in reducing smoking prevalence is inconclusive 13, we conducted a sensitivity analysis to assess the robustness of our models to the inclusion of NRT advertising. The NRT GRP data we used covered the United Kingdom rather than England each month from January 2002 to December 2009; GRPs for England could not be separated from those for the other countries of the United Kingdom. We re‐ran all final models and included NRT GRPs for the month of the OS interview, 1 month and 2 months later as three separate cubic regression splines (Pearson correlation coefficient in all two‐way comparisons was less than 0.17, suggesting little correlation between terms).

### Predicting the impact of GRPs

We used the fitted models to investigate what impact GRPs had on cigarette consumption and smoking prevalence during the study period. With the original data, we predicted cigarette consumption for individuals in 2002 and 2009 and computed the percentage reduction in the model‐predicted yearly means (2009 was used rather than 2010, so that we had individuals being interviewed throughout the year). To predict the percentage reduction had there been no TC advertisements during the time‐period, we repeated this procedure but set GRPs = 0 in the original data set. Similarly, the difference in annual smoking prevalence predicted for 2002 and 2009 was compared between the original GRP data and with GRP = 0.

## Results

**Figure 1:**
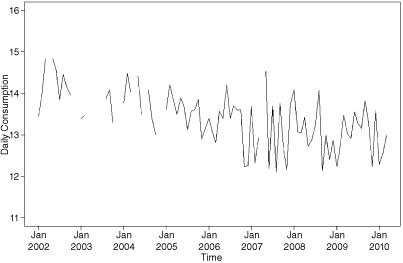
Time–series plot of average cigarette consumption in England per month from February 2002 to April 2010. Gaps indicate periods when no Opinions and Lifestyle Survey (OS) data were collected

During the period covered by the study, average daily consumption and smoking prevalence has been declining and TC efforts have increased, with both the tobacco control score and weighted average price of a packet of 20 cigarettes increasing over time (Figs 1). GRPs, in contrast, are characterized by peaks and troughs with no clear secular trend (Fig. 3).

**Figure 2:**
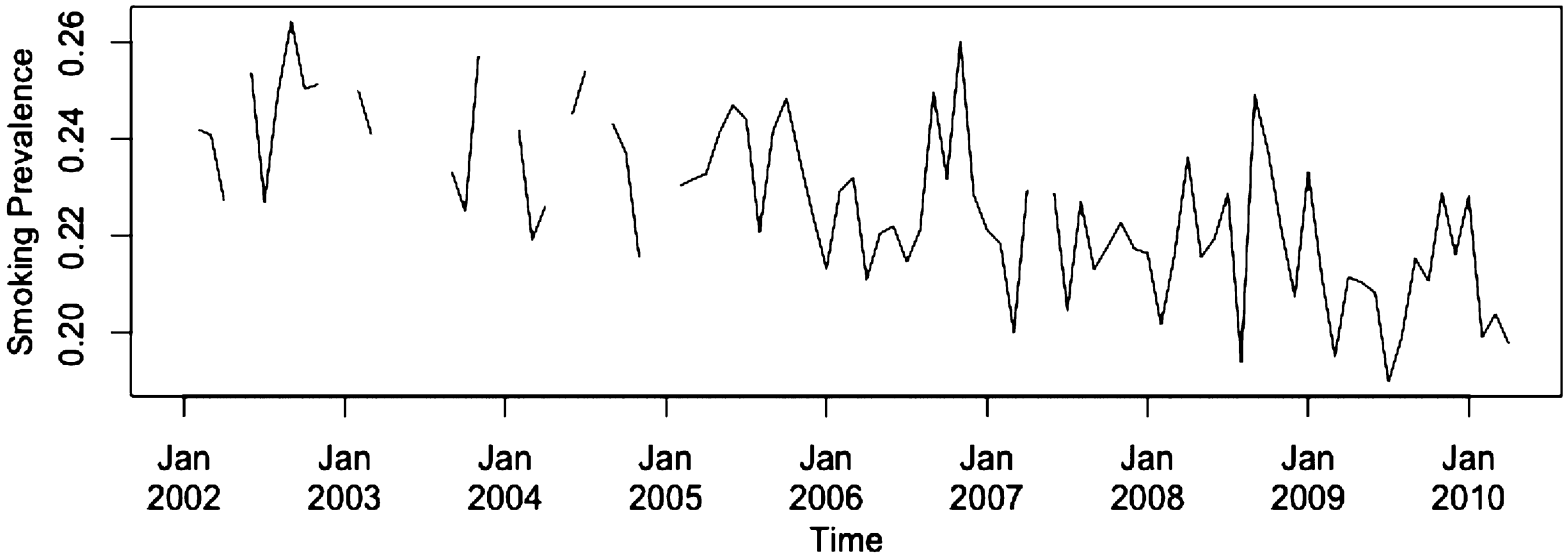
Time–series plot of smoking prevalence in England per month from February 2002 to April 2010. Gaps indicate periods when no Opinions and Lifestyle Survey (OS) data were collected

**Figure 3:**
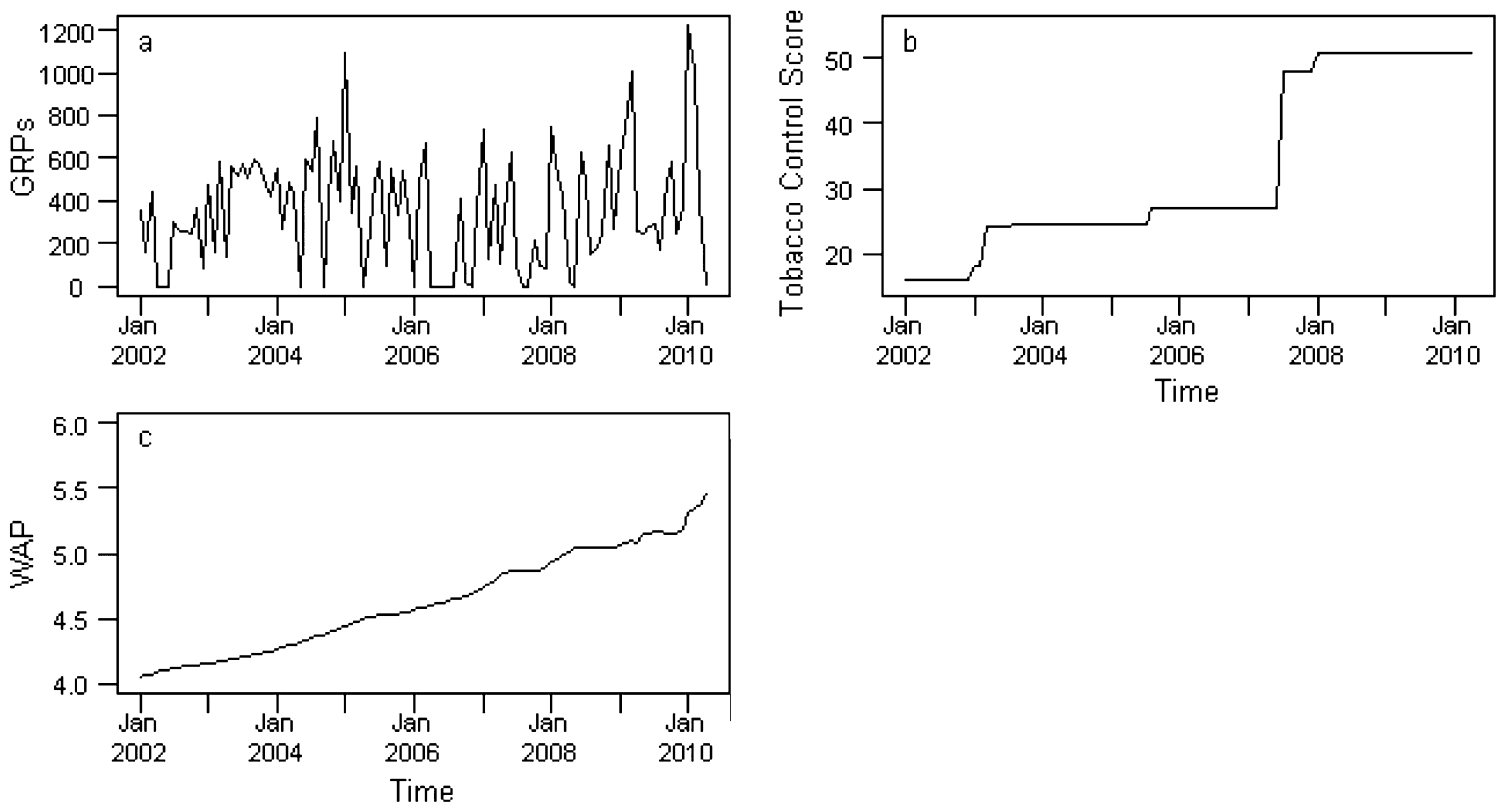
Time–series plots of (a) gross rating points (GRPs), (b) tobacco control score and (c) weighted average price (WAP) of a packet of 20 cigarettes

Over 84 months between February 2002 and April 2010, inclusive, 19 488 and 81 256 adults were included in the consumption and smoking prevalence analyses, respectively. The smooth terms for GRPs at 1 month and 2 months previous were found to be linear (EDF = 1) in both the smoking prevalence and consumption models, and were replaced with linear terms.

### Smoking prevalence

After controlling for other TC strategies and cigarette costliness, as well as individual characteristics, smoking prevalence was associated with GRPs 2 months previous (Table 1). A 400‐point increase in GRPs, equivalent to all adults in the population seeing four advertisements per month, was associated with 3% lower odds of smoking [odds ratio (OR) of 0.97, 95% confidence interval (CI) = 0.95, 0.999] 2 months later. The model predicted that smoking prevalence fell by 3.7 percentage points between 2002 and 2009. Had there been no TC television advertisements during this time‐period, the model predicted that prevalence would have fallen around 3.2 percentage points. This 0.5 percentage‐points fall in smoking prevalence attributable to MMCs represents 13.5% of the percentage‐points decline over this period and equates to an estimated 193 000 fewer smokers in England over this 7‐year period, or approximately 27 500 per year.

**Table 1:** Results of regression analysis to detect an association between tobacco control gross rating points (GRPs) and smoking behaviour.^a^

Effects^c^	Average weekly consumption				Smoking prevalence			
	% change	(95% CI)	EDF	P^b^	OR	(95% CI)	EDF	P^b^
Parametric terms:								
GRPs 1 month earlier	−1.80	(−3.11, −0.47)		<0.01	0.98	(0.95, 1.01)		0.13
GRPs 2 months earlier	0.20	(−1.14, 1.56)		0.78	0.97	(0.95, 0.999)		0.04
Smooth terms:								
GRPs (immediate effect)			1.88	0.13			1.6	0.49

^a^GRPs at different lags were initially considered as non‐linear terms and if they were found to be linear [effective degrees of freedom (EDF) = 1] then replaced with linear terms. The table presents the linear effects first, with a point estimate, 95% confidence interval (CI) and *P*‐value for the percentage change in consumption and odds ratios (ORs) for smoking prevalence associated with a 400‐point increase in GRPs. For the non‐linear effects it is not possible to present a single point estimate, as this varies depending on the value of the variable. The table reports the estimated degrees of freedom, which is a measure of how ‘wiggly’ or non‐linear the term is (EDF = 1 corresponds to a straight line; that is, a linear effect) plus *P*‐value. ^b^*P*‐value from a *t*‐test on parametric regression coefficients and *F*‐test on smooth terms. ^c^Regression models also include cubic regression splines for age, income and cigarette costliness, linear term for number of adults in the household and categorical variables for gender, social class, education, employment status and government office region of residence.

### Consumption

After controlling for other TC strategies, cigarette costliness and individual characteristics, average consumption was associated with GRPs in the previous month (Table 1). A 400‐point increase in TC advertisement GRPs was associated with a statistically significant 1.80% (95% CI = 0.47, 3.11) decline in average consumption in the following month. The model predicted that average consumption fell by 10.7% between 2002 and 2009 and that without TC television advertisements this fall would have been 9.5%. In other words, 1.2/10.7 (i.e. 11.2%) of the estimated decline in cigarette consumption could be attributed to TC MMCs.

The sensitivity analysis using NRT GRPs did not change our results, and we found no effect of NRT GRPs on either smoking prevalence or consumption (*P* > 0.1 for all smooth terms).

## Discussion

We found a small but statistically significant association between GRPs and both cigarette consumption and smoking prevalence. After adjustment for other TC policies, cigarette costliness and individual characteristics, a 400‐point increase in GRPs was associated with a 3% lower odds of smoking (OR = 0.97) 2 months later and a 1.80% reduction in average consumption in the following month. The association between GRPs and smoking prevalence was only just statistically significant at the 5% level, but is supported by research in Australia 13 which found a similar small association between smoking prevalence and TC GRPs in the 2 months previous. In the Australian study, a time–series analysis of aggregate monthly smoking prevalence over an 11‐year period, a 390‐point increase in GRPs was significantly associated with a 0.3 percentage‐point drop in adult smoking prevalence 13. This close link between behavioural change and recent media exposure is consistent with findings observed in England 23 and Australia 29 on their effectiveness in triggering quit attempts.

### Limitations

Although we adjusted for all the known major factors that determine tobacco use (individual characteristics, cigarette costliness and other TC policies), we cannot completely rule out unmeasured confounding which could have occurred via unmeasured, unknown variables that confound the relationship between GRPs and smoking outcomes or via imperfect measurement of the major factors included in our analysis.

We used population‐level data for GRPs and weighted average price (WAP), and our model assumes that there is no individual‐level variability. In reality, GRPs measure average potential exposure; individual‐level exposure will vary depending on frequency of actual television viewing and attention to advertisements. Similarly, WAP does not reflect the brand smoked by an individual and we know that price trends have varied markedly by brand 31. The proportion of respondents who did not report income increased from approximately 8% in 2002 to 14% in 2010. However, any potential bias this might cause if this varies by age, gender or SES would have been corrected for by inclusion of these variables in the models. Although the number of months per year that OS surveys were conducted varied, as described in the Methods, the months in which there are no surveys is not expected to be correlated with GRPs and therefore should not introduce any bias in the findings. Finally, the OS survey does not release survey design variables and we were therefore unable to adjust standard errors for the clustering in the survey design.

### Strengths

This is the first study in Europe to look at the effect of TC advertisements on smoking prevalence and consumption and the first international study to examine impacts on consumption in an environment of intense TC activity. In contrast to the aforementioned study in Australia 13, our use of individual‐level data avoids ecological fallacy, where individual behaviour is inferred from aggregated data 32. Furthermore, this study uses a statistical method which allows for non‐linear effects of the explanatory variables, including GRPs. This is in contrast to most other studies looking at the impact of MMCs on smoking behaviour which assume linear effects of GRPs. GAMs are a suitable approach for analysing time–series data when we want to model the non‐linear effects of covariates and allow residuals to have non‐normal distributions (for example, Poisson). It can also be used on data collected at unequal intervals in time. Autoregressive integrated moving average (ARIMA) models are not appropriate here because the data would have to consist of a single time–series of equally spaced and ordered time‐points, spline terms cannot be included in the models and residuals are assumed to be normally distributed.

### Implications for future MMCs

The population impact of a TC intervention is measured in terms of effectiveness × reach. While the effect of a mass media intervention per smoker may be small, it has the potential to reach a large proportion of smokers and thus to have a significant population impact. Therefore, although the effect sizes estimated here are small, they translate to an appreciable impact at the population level. We estimate that, over the period 2002–09, 11.2% of the decline in cigarette consumption and 13.5% of the decline in prevalence was attributable to the impact of MMCs. The decline in smoking prevalence observed via our model equates to approximately 27 500 fewer smokers per year, and thus to appreciable health gain. Government expenditure on television advertising campaigns from January 2002 to December 2009 was £78 million, equating to £406 per additional non‐smoker based on our model predictions. Importantly, this figure considers only the direct impact of MMCs on smoking prevalence. Evidence suggests that MMCs might have indirect effects 33, for example, by changing social norms against smoking and in favour of TC policies which may increase sensitivity to other TC policies (thereby further reducing prevalence) and increase the likelihood of such policies being implemented. While comparisons with the NHS Stop Smoking Services (SSS), which have only direct impacts on smokers, may therefore be limited, they show that MMCs are at least as cost‐effective. The SSS cost approximately £950 per successful 1‐year quitter based on the total cost of SSS between April 2002 and March 2010 34 and number of biochemically verified 4‐week quitters using SSS and the proportion expected to relapse after 1 year 35. In reality, MMCs and SSS are likely to be interrelated and probably reach smokers with different levels of dependence.

Although the majority of campaign exposure for the population is still on television, the media environment changed during the study period (for example, an increasing number of UK adults are using the internet for viewing catch‐up television services, the number of cable channels has increased and viewers have the ability to fast‐forward through television advertisements) and will continue to evolve. As such, the reach of these campaigns may have reduced over time. Research on the most efficient use of various media types in this changing environment, refining media message and intensity and the cost‐effectiveness of campaigns are research goals for the future.

In conclusion, TC television advertising is associated with reductions in smoking prevalence and consumption, even in a jurisdiction with comprehensive TC policies.

### Declaration of interests

None.

## Supplementary Material

**Box S1** Weighted average price of cigarettes.
**Figure S1** Time‐line of tobacco control policies in England, 2000–10.
**Table S1** Coding scheme for tobacco control policies.
**Table S2** Predictors included in generalized additive models.
