# Supplementary material for: Effectiveness of tobacco control television advertising in changing tobacco use in England: a population‐based cross‐sectional study
Source: Addiction. 2014 Mar 10;109(6):986–94. doi: 10.1111/add.12501 (PMC4114556; doi:10.1111/add.12501)
Supplement: Supplementary file 1 — Box S1 Weighted average price of cigarettes. Figure S1 Time‐line of tobacco control policies in England, 2000–10. Table S1 Coding scheme for tobacco control policies. Table S2 Predictors included in generalized additive models. [file add-109-986-s4.docx]

**Effectiveness of tobacco control television advertising in changing tobacco use in England: a population-based cross-sectional study**

Michelle Sims, Ruth Salway, Tessa Langley, Sarah Lewis, Ann McNeill, Lisa Szatkowski, Anna B Gilmore

**Online Supporting information**

**Figure S1. Timeline of tobacco control policies in England, 2000-2010**

**
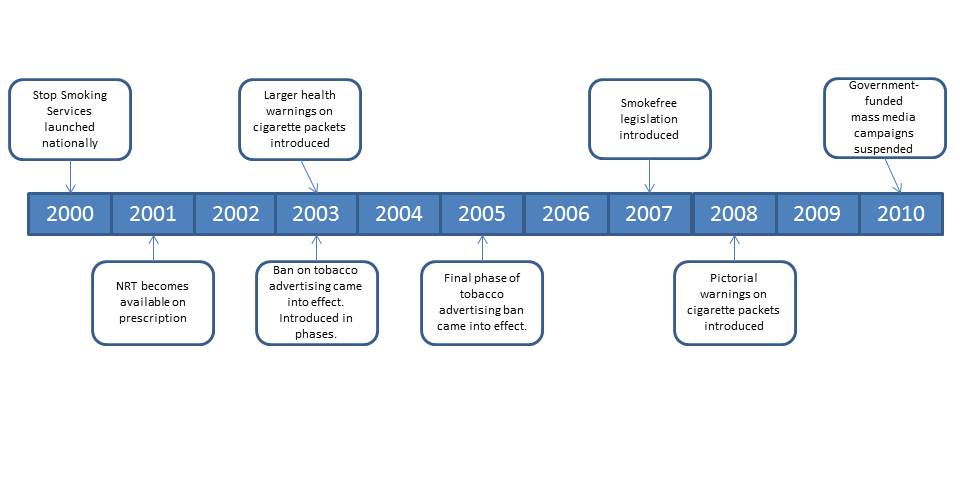
**

**The Tobacco Control Score**

The Tobacco Control Score was based on 4 policies: (1) smokefree work and public places, (2) bans on advertising and promotion, (3) health warning labels on cigarette packets and (4) treatment to help smokers stop. Scoring for each policy was identical to that assigned by the Tobacco Control Scale (TCS), developed by Joossens and Raw (2006)([1](#_ENREF_1)) to compare tobacco control efforts across Europe. Cigarette pricing, based on the price of Marlboro and the Most Popular Price Category (MPPC), and spending on public information campaigns are also included in the TCS, but were excluded from our coding scheme as they were dealt with separately. Price was measured differently using the weighted average retail selling price (WAP) of cigarettes (Box S1 in Supporting material), which more accurately reflects price trends than the MPPC,([2](#_ENREF_2)) and in January 2011, replaced MPPC as the method for calculating tobacco excise levels in all European Union Member States.([3](#_ENREF_3)). Public information campaigns were also considered separately from other tobacco control policies as they form the focus of this study and were measured via GRPs rather than spending. Information on the date and nature of policies implemented in England was obtained from an extensive literature search and informal discussions with tobacco control experts.

**Table S1. Coding scheme for tobacco control policies (a shortened version of the Tobacco Control Scale in Europe^1^)**

| **Smoke free work and other public places** | **22** |
| --- | --- |
| **Workplaces excluding cafes and restaurants – one only of** | **10** |
| Complete ban without exceptions (no smoking rooms); enforced | 10 |
| Complete ban, but with closed, ventilated, designated smoking rooms; enforced | 8 |
| Complete ban, but with ventilated, designated smoking rooms; enforced | 6 |
| Meaningful restrictions; enforced | 4 |
| Legislation, but not enforced | 2 |
| **Cafes and restaurants – one only of** | **8** |
| Complete ban; enforced | 8 |
| Complete ban, but with closed, ventilated, designated smoking rooms ; enforced | 6 |
| Meaningful restrictions; enforced | 4 |
| Legislation, but not enforced | 2 |
| **Public transport and other public places – additive** | **4** |
| Complete ban in domestic trains without exceptions | 1 |
| Complete ban in other public transport without exceptions | 1 |
| Complete ban in educational, health, government and cultural places without exceptions | 2 |
| OR Ban in educational, health, government and cultural places, but with designated | 1 |
| smoking areas or rooms |  |
| **Comprehensive bans on advertising and promotion (points for each type of ban - additive)** | **13** |
| Complete ban on tobacco advertising on television | 3 |
| Complete ban on outdoor advertising (eg. posters) | 2 |
| Complete ban on advertising in print media (eg. newspapers and magazines) | 2 |
| Complete ban on indirect advertising (eg. cigarette branded clothes, watches, etc) | 2 |
| Ban on point of sale advertising | 1 |
| Ban on cinema advertising | 1 |
| Ban on sponsorship (0.5 for domestic and 0.5 for international) | 1 |
| Ban on internet advertising | 0.5 |
| Ban on radio advertising | 0.5 |
| **Large direct health warning labels** | **10** |
| **Rotating health warnings** | **2** |
| **Size of warning – one only of** | **4** |
| 10% or less of packet | 1 |
| 11 – 25% of packet | 2 |
| 26 – 40% of packet | 3 |
| 41% or more of packet | 4 |
| **Contrasting colour (eg. black lettering on white background)** | **1** |
| **Pictorial health warnings** | **3** |
| **Treatment to help dependent smokers stop** | **10** |
| **Quitline – one only of** | **2** |
| Well funded national quitline or well funded quitlines in all major regions of country | 2 |
| OR National quitline with limited funding or a patch work of small local quitlines | 1 |
| **Network of smoking cessation support (3) and reimbursement of treatment (3)** | **6** |
| Cessation support network covering whole country (3); free (3) | 6 |
| Cessation support network, but only in selected areas, eg. major cities (2); free (3) | 5 |
| Cessation support network covering whole country (3), partially free (2) | 5 |
| Cessation support network, but very limited, just a few centres (1), free (3) | 4 |
| Cessation support network, but only in selected areas, eg. major cities (2), partially free (2) | 4 |
| Cessation support network covering whole country (3), not free (0) | 3 |
| Cessation support network, but very limited, just a few centres (1), partially free (2) | 3 |
| Cessation support network, but only in selected areas, eg. major cities (2); not free (0) | 2 |
| Cessation support network, just a few centres (1), not free (0) | 1 |
| **Reimbursement of medications – one only of** | **2** |
| Reimbursement of pharmaceutical treatment products | 2 |
| OR Partial reimbursement of pharmaceutical treatment products | 1 |
| **MAXIMUM POSSIBLE SCORE** | **55** |

^1^ Joossens L, Raw M. The Tobacco Control Scale: a new scale to measure country activity. *Tobacco Control* 2006;**15**:247-53.

**Box S1. Weighted average price of cigarettes**

Weighted average price was calculated as:

$\sum_{i=1}^{n} m_{i}p_{i}$ (Eqn 1)

where

*p_i_* is the price of cigarette brand *i*, *m_i_* is the volume market share of cigarette brand *i* and n is the number of cigarette brands.

Cigarette price data were obtained for packs of 20 cigarettes (or 19 cigarettes for brands sold only in 19s). There is no single source of price data available for the whole study period. From 2002 to 2005, we obtained recommended retail prices of all major cigarette brands for the UK market from PriceChecker, a supplement of the weekly magazine ‘Retail Newsagent’ published in the UK. We used June data (published in May) and December data (published in November) from PriceChecker, except in 2005 when October data were used (PriceChecker ceased publication in October 2005). From November 2006, Nielsen began to publish cigarette sales data, including price and volume (the number of cigarette packs sold for each brand), for the Great Britain market. We obtained 7 monthly data between November 2006 and May 2008 (thus including November 2006, May and November 2007 and May 2008) and monthly data from November 2008 to December 2011. The expense of Nielsen data prevented us obtaining monthly data before November 2008. A comparison between the two data sources indicated that PriceChecker and Nielsen could provide comparable price data over time.([2](#_ENREF_2))

Volume market share was obtained from the General Household Survey (GHS) for 2002 to 2005 and Nielsen from 2006 to 2010 (as detailed above). GHS, an annual survey designed to be representative of the population of Great Britain, asks smokers aged 16 years and over which brand of cigarettes they smoke and how many sticks they smoke a week. These questions were used to calculate market share by brand.([2](#_ENREF_2))

An exploratory analysis of trends in WAPs from November 2008 until December 2011 (the time period when monthly data were available) indicated it was reasonable to derive a WAP for months when cigarette price and volume were unavailable by linearly interpolation.

**Table S2. Predictors included in generalised additive models**

| **Predictor** | **Description** |
| --- | --- |
| Tobacco control score | Categorical term for tobacco control score for England. 7 categories (16, 18, 24, 24.5, 27, 48, 51)* |
| Cigarette costliness | Weighted average price of packet of 20 cigarettes in month of interview divided by average monthly gross income of respondent (i.e. proportion of monthly income that a packet of cigarettes costs). Include as cubic regression spline. |
| Number of adults in the household | Number of adults in households of OS respondent. Include as linear term. |
| Age | Age of OS respondent. Include as cubic regression spline. |
| Gender | Categorical term for gender of OS respondent |
| Government office region | Categorical term for government office region of residence of OS respondent. 9 categories: East Midlands, East of England, London, North East England, North West England, South East England, South West England, West Midlands and Yorkshire & the Humber |
| Social class | Categorical term for national statistics socio-economic classification (NS-SEC) of OS respondent. 4 categories: managerial and professional occupations, intermediate occupations, routine and manual occupations, not classified. |
| Employment | Categorical term for employment status of OS respondent. 3 categories: employed, unemployed, economically inactive. |
| Education | Categorical term for highest level of qualification that OS respondent has received. 8 categories: Degree level qualification (or equivalent), Higher educational qualification below degree level, A-Levels or Highers, ONC / National Level BTEC, O Level or GCSE equivalent (Grade A-C) or O Grade/CSE equivalent (Grade 1) or Standard Grade level 1-3, GCSE grade D-G or CSE grade 2-5 or Standard Grade level 4-6, Other qualifications (including foreign qualifications below degree level) , No formal qualifications. |
| Income | Total gross income from all sources before deductions for income tax, National Insurance etc. Include as a cubic regression spline. |

*Scores increased during the study period, starting with a score of 16 in January 2002, then changing with each introduction or change in a tobacco control policy and reaching 51 by January 2008. Including this as a categorical term means we are looking at changes in the outcomes when a tobacco control policy is introduced or changed rather than assessing the impact of a change in magnitude of the score itself.

**References**

1. Joossens L, Raw M. The Tobacco Control Scale: a new scale to measure country activity. Tob Control. 2006;15(3):247-53.

2. Gilmore AB, Tavakoly B, Taylor G, Reed H. Understanding tobacco industry pricing strategy and whether it undermines tobacco tax policy: the example of the UK cigarette market. Addiction. 2013;108:1317-26.

3. Council Directive 2011/64/EU of 21 June 2011 on the structure and rates of excise duty applied to manufactured tobacco 2011. Available from: <http://eur-lex.europa.eu/LexUriServ/LexUriServ.do?uri=OJ:L:2011:176:0024:0036:EN:PDF>.
